# The Role of Nanoscale Seed Layers on the Enhanced Performance of Niobium doped TiO_2_ Thin Films on Glass

**DOI:** 10.1038/srep32830

**Published:** 2016-09-09

**Authors:** Stefan Nikodemski, Arrelaine A. Dameron, John D. Perkins, Ryan P. O’Hayre, David S. Ginley, Joseph J. Berry

**Affiliations:** 1 Department of Metallurgical and Materials Engineering, Colorado School of Mines, 1500 Illinois Street, Golden, CO 80401, USA; 2 National Renewable Energy Laboratory, 15013 Denver W Pkwy, Golden, CO 80401, USA

## Abstract

Transparent conducting oxide (TCO) coatings with decreased cost and greater process or performance versatility are needed for a variety of optoelectronic applications. Among potential new TCO candidates, doped titanium dioxide is receiving particular interest. In this study, niobium-doped titania bilayer structures consisting of a nanoscale seed layer (deposited by atomic layer deposition or RF magnetron sputtering) followed by a thick bulk-like layer were grown directly on glass in order to examine the effects of the seed layer processing on the subsequent crystallization and electrical properties of these heterostructures. Observations from Raman spectroscopy suggest that higher oxygen content in the seed layer suppresses the formation of detrimental titania polymorph phases, found in films produced by annealing directly after synthesis without any exposure to oxygen. Furthermore, our results indicate that the generation of excellent Nb:TiO_2_ conductors on glass (without breaking vacuum) only occurs within a narrow processing range and that the sequential deposition of oxygen-poor layers on oxygen-rich layers is a critical step towards achieving films with low resistivity.

New high-performance yet low-cost transparent conducting oxide (TCO) contacts are needed for high-efficiency optoelectronic applications. Many of these applications rely on indium-based TCOs, most commonly indium tin oxide, or ITO. There are, however, concerns regarding the abundance of indium and resulting material reserves as it relates to future ITO availability and costs^1^,^2^,^3^,^4^,^5^. Anatase titania (TiO_2_) has been shown to be a viable candidate for TCO applications^6^,^7^,^8^. Upon doping with niobium (optimally between 3–6 at.%)^6^,^7^,^8^, titania shifts from non-metal to metallic-like properties resulting in both high electrical conductivity and high optical transmittance. For anatase TiO_2_ thin films with Nb doping in this ideal range deposited on single crystal substrates, the resistivity is comparable to that of conventional ITO films^6^,^7^,^8^,^9^.

Presently, the best Nb-doped TiO_2_ (TNO) films have been achieved by physical vapor deposition on epitaxially-matched single crystal substrates such as LaAlO_3_ and SrTiO_3_^6^,^7^,^8^,^9^. Generally, studies involving TNO films grown on these high quality substrates (with subsequent high temperature anneals in reducing atmospheres) result in both high conductivity (in some cases ~3000 S/cm) and moderate transparency (60–80% for films ~200 nm thick)^6^. For films on these substrates, the epitaxial relationship between the substrate and the doped titania stabilizes the high mobility anatase phase while excluding the lower mobility rutile phase^6^,^7^,^10^,^11^. However, large-scale production of TCO films on crystalline substrates is costly and technologically limiting, thus alternative substrate choices (such as glass) are more attractive for a broader range of industrial applications. Recently, significant efforts have been undertaken to translate these results from crystalline substrates to non-epitaxial systems^7^. Studies of TNO films deposited directly on glass have concluded that the most crucial growth parameters influencing the final crystalline phase are deposition/annealing temperature and gas pressure/composition^12^,^13^. However, only samples subjected to harsh annealing conditions (high temperatures of ~600 °C in pure H_2_) produce the most conductive and transparent films. This high temperature reducing anneal is required to produce larger crystalline domains (thus reducing grain boundary scattering), which is made difficult by the amorphous nature of the substrate. One method to improve the crystal growth of oxides is to use seed/buffer layers to act as an intermediary phase between the film and the amorphous substrate surface^14^. Recently, several studies investigating the effect of nanoscale seed layers on the growth orientation of pure and doped TiO_2_ on glass have been conducted^15^,^16^,^17^,^18^,^19^,^20^. Yang *et al.* showed that a bilayer SrTiO_3_/TiN template grown by physical vapor deposition on glass substrates produces anatase thin films with uniaxial c-axis orientation^15^. Hoang *et al.* reported some promising results, whereby low temperature sputter deposition of an initially amorphous oxygen-rich Nb-TiO_x_ film followed by a thicker oxygen-deficient Nb-doped TiO_x_ film with a subsequent rapid thermal treatment produced structures with high conductivities (~1000 S/cm)^16^. While this approach has achieved conductivities close to ~1000 S/cm, this method has yet to rival the epitaxially grown materials. In addition, it is still unclear what the impact of doping concentration in the TiO_2_ layer has on the phase stability. Shibata *et al.*^17^,^18^, Yamada *et al.*^19^ and Taira *et al.*^20^ have conducted a significant amount of research utilizing Ca_2_Nb_3_O_10_ nanosheets with a 2D perovskite structure to template the growth of high quality (001) oriented anatase films, which in some cases (when combined with PVD deposited Nb-doped TiO_2_ films) have succeeded in significantly enhancing the carrier transport properties.

In this study, a design-of-experiments matrix based on a survey of reported results was developed to gain insight regarding the key deposition parameters along with resulting physical properties of the seed and bulk layers (structural phase, dopant concentration, crystallinity, oxygen content, and annealing time) ability to control desirable TNO film properties leading to desirable electronic properties (high carrier concentration and mobility) leading to high electrical conductivity. The seed layers were fabricated using both atomic layer deposition (ALD) and RF magnetron sputtering. ALD TiO_2_ seed layers were deposited at low and high temperature with various thicknesses using two different titanium precursors (TiCl_4_, Ti[OCH(CH_3_)_2_]_4_) and two different oxygen sources (H_2_O, H_2_O_2_). Sputtered TNO seed layers of varying thickness and oxygen content were deposited immediately prior to the deposition of the bulk TNO film in the same chamber without breaking vacuum. The bulk layer film had a constant thickness of ~140 nm. After deposition, the seed + bulk layer films were annealed at various temperatures under reducing atmospheres (both *in-situ* and *ex-situ*), to clarify the effect of post deposition oxygen exposure and annealing temperature on film properties. For specific procedural conditions see Table 1.

It is believed that the stabilization of the anatase phase in the seed layer (which subsequently acts as a nucleation center for the bulk film crystallization during annealing) is critical to achieve high conductivity films. We confirm this by demonstrating that both ALD and sputter seed layers can form stable anatase seeds on glass that enables subsequent crystallization of the overlying layer at around 300 °C. Further heating causes the activation of the niobium dopant, releasing additional electronic carriers into the conduction band. This seed layer + overlying layer strategy results in films with vastly superior electrical properties compared to monolithic films deposited directly on glass (i.e., without a seed layer) under equivalent conditions (~2–3 fold improvement). Furthermore, our results suggest a key guiding principle to achieve films possessing high phase purity and high electrical conductivity is related to the oxygen content in the underlying seed layer. Only by depositing sputtered seed layers in oxygen rich environments can the formation of detrimental titania polymorph phases be suppressed.

## Results

### *In-situ* XRD annealing

**Figure 1:**
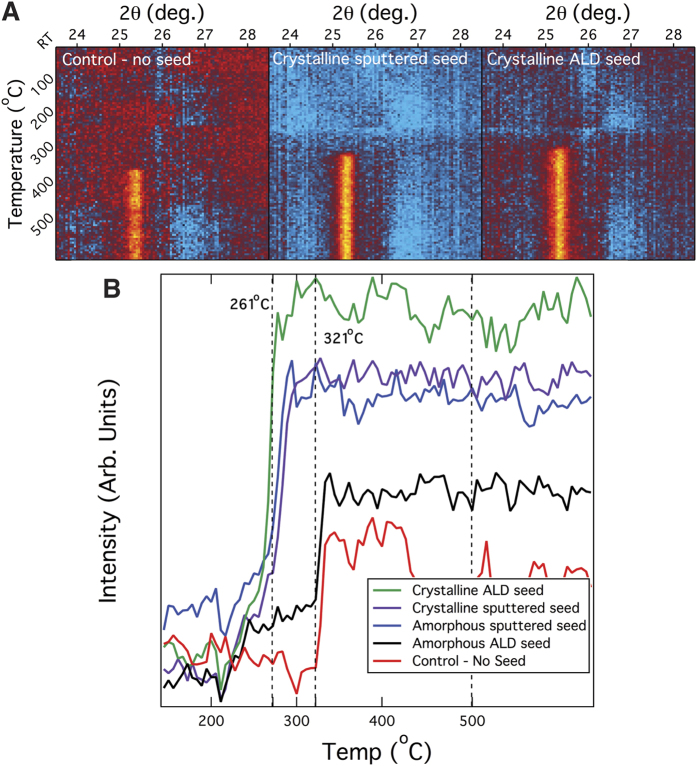
(**A**) Magnification of the 25.5^o^ anatase x-ray peak for Nb:TiO_2_ films deposited on seed layers as well as directly on glass. (**B**) Temperature dependence of Nb:TiO_2_ film phase formation (produced from the average integrated intensity of all anatase x-ray peaks).

To understand the influence of the seed layer on the crystallization of the Nb doped TiO_2_ film we performed a series of *in situ* XRD annealing experiments. Figure 1 plots the x-ray intensity (in the 25.5° anatase peak region) as a function of both 2θ and substrate temperature for 140 nm thick TNO films deposited on 30 nm thick ALD and sputtered TNO seed layers as well as a control sample consisting of an 140 nm thick TNO film deposited directly on the glass substrate without a seed layer. For complete scan information with standard x-ray reference patterns for both anatase and rutile phases, see supplementary information (Figure S1).

The fluctuations in the x-ray intensity (observed most prominently in the 20–35° range) are the result of background subtraction. The Anton Paar DHS 900 dome used to encapsulate the samples (and trap the N_2_ purge gas) has a very intense x-ray signature, and the associated peaks are difficult to completely eliminate. Nevertheless, diffraction patterns were recorded as Debye ring patterns in a two-dimensional detector image, and the XRD results after background corrections clearly indicate that randomly-oriented polycrystalline TiO_2_ films were obtained. All samples (including the control) were found to be anatase phase for our set of annealing conditions (supplementary – Figure S1). One clear distinction between the seed-based samples and the control sample is the temperature at which the onset of crystallization occurs (Fig. 1A). In order to better visualize the temperature onset of crystallization, the integrated intensity of all anatase peaks was calculated, averaged, and plotted as a function of temperature in Fig. 1B. TiO_2_ crystallization for all samples, regardless of seed properties, occurred in the temperature range of 260–320 °C and is concluded within a short time span (30 minutes). The presence of crystalline ALD seed layers and both crystalline and amorphous oxygen rich sputtered seed layers produced films that crystallized at the low end of this temperature range (260 °C) while the absence of a seed layer in the control sample delayed crystallization to the upper end of this temperature range (330 °C). The negligible difference in the crystallization temperature of a thicker TNO layer with an underlying oxygen rich sputtered seed layer (crystalline or amorphous) may indicate that a high oxygen content in the seed layer leads to the reduction of the nucleation energy barrier. On the other hand, initially amorphous ALD seed layers (deposited at low temperatures ~100 °C) clearly do not lead to a reduction in this energy barrier for film crystallization, as these films have a similar crystallization onset as the control sample. Another key observation from these experiments is the variation in the average integrated x-ray intensity despite all films being the same thickness. For samples with sputtered seeds (whether crystalline or amorphous), the average integrated intensity is relatively similar, and it was found that the electrical properties of the sputtered samples were also comparable. This suggests that the final crystallite sizes are approximately equal and lead to near identical rates of electron scattering. However, TNO films deposited on crystalline versus amorphous ALD seed layers resulted in markedly different behavior. Compared to their amorphous ALD counterparts, TNO films deposited on crystalline ALD seed layers exhibited a much higher average integrated intensity upon annealing (see Fig. 1) and growth oriented along particular crystal planes (see supplementary Figure S1 – compare “crystalline ALD seed” with “no seed”). The PVD deposited samples also showed preferred orientation compared to the control sample, particularly the lower relative intensity of the (004) (105), and (211) planes in favor of more intense (101) and (200) crystal planes.

### Raman mapping

**Figure 2:**
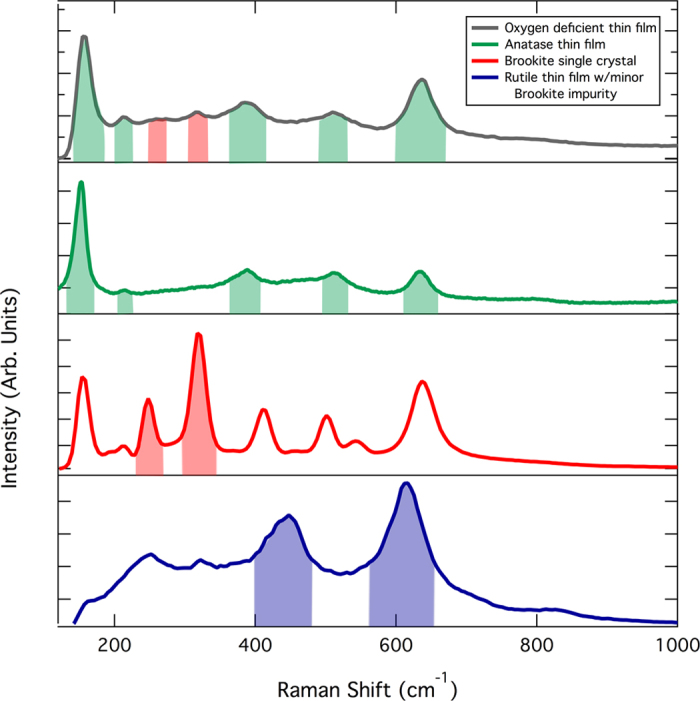
Raman spectra for various TiO_2_ polymorph samples. The highlighted regions provide an example of the integrated peak intensity used to calculate the impurity ratios for bulk films. Note, these spectra are presented to highlight the regions of interest and have not been subjected to any background subtraction (which is required for the calculation of the anatase/impurity ratio).

**Figure 3:**
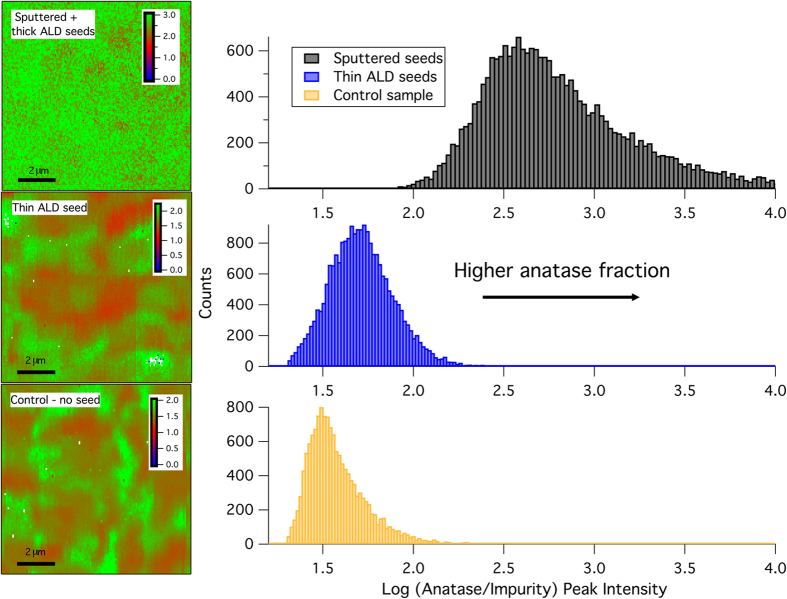
Raman mapping results for Nb:TiO_2_ films. Maps are plotted on a log scale. Histogram of anatase fraction values for a variety of seed layer samples and the control.

Due to the low spatial resolution of our 2D x-ray detector, Raman spectroscopy using a confocal microscope (100x objective) and a Nd^3+^:YAG laser with an operational wavelength of 532 nm was additionally used to spatially resolve the crystal phases of the annealed heterostructures. Additionally, Raman spectroscopy is also very sensitive to minor impurity phases present in our films that otherwise might go undetected by XRD. Spatial maps of the films measuring 10 microns × 10 microns were constructed by combining sequential line scans across the sample. In our case, only XY information (resolution ~250 nm) is available and the measured phase at a given position represents an average through the entire film thickness. Both anatase and brookite polymorphs are observed in our films by Raman spectroscopy. The observation of brookite in our films is surprising since rutile is the more commonly observed and thermodynamically stable TiO_2_ polymorph. We know that a number of other polymorphic oxides have metastable bulk phases (gamma vs. alpha alumina, iron oxide compounds)^21^. The polymorphs that are often metastable at the bulk scale often predominate at the nanoscale due to thermodynamic considerations (metastable bulk phase may have a lower surface energy). Therefore, there is a crossover in the energetics when these phases are present at the nanoscale. It is possible that titania is such a material where the metastable brookite phase may be present as nanoparticles imbedded in a primarily anatase matrix. Nevertheless, the presence of the brookite polymorph in our films is strongly reinforced by direct comparison to Raman measurements of single crystal brookite mineral samples using the same system (Fig. 2). The relative intensity of the anatase and brookite signals can be deconvoluted (see Fig. 2) by modeling each spot scan with a linear combination of pure phase spectra (with different weighting factors). By taking a ratio of the two signals, we can estimate the anatase to brookite fraction. These values are plotted in Fig. 3.

Because of the small percentage of brookite in the films, each graph is plotted on a log scale to better resolve contrast between regions with “high” to “low” brookite content. Thus, a value of 0.0 would represent a 50/50 mixture between the brookite and anatase phases; a value of 1.0 would be a 10:1 ratio of anatase to brookite and so on. Each sample was annealed in vacuum to a maximum temperature of 540 °C for 2 hrs immediately following deposition and subsequently mapped. A histogram of the intensity ratios was generated for each sample and is included beside each corresponding Raman map.

The control sample (TNO deposited directly on glass) has mixed phase behavior where the ratio of the weighting factors (between the anatase and brookite phases) in the Raman spectra deconvolution ranges from 10–100:1 (closer to 30:1 given the histogram peak value of ~1.5). TNO films deposited on thinner (~5 nm) ALD seed layers show very similar post-anneal structure to the control sample. However, the anatase-to-brookite ratio has a slightly higher average value (50–60:1) in this case, which is indicated by a shift in the histogram peak to greater values. TNO films deposited on thick (30 nm) ALD seed layers as well as on oxygen-rich sputtered seed layers achieve the greatest degree of anatase fraction. In these cases, the brookite fraction is extremely low and is only just above the background signal. The majority of the film has an anatase to brookite ratio on the order of 100–1000:1 (with some regions achieving even higher fractions). The thickness of the sputtered seeds has little impact on the anatase-to-impurity ratio as observed by Raman mapping. The more important deposition parameter affecting this ratio is the oxygen content of the seed. We found that the brookite phase is suppressed as long as the underlying seed layer has high oxygen content (see supplementary Figure S2). One critical difference between the Raman and XRD experiments was the exposure to the ambient environment. XRD samples were necessarily exposed to ambient air before the experiment commenced whereas Raman mapping specimens were annealed in the vacuum chamber directly following deposition. Therefore, short-term O_2_ exposure could have resulted in changes to the nature of the phases present post-annealing.

Atomic force microscopy (AFM) and field emission scanning electron microscopy (FESEM) were used to evaluate the smoothness and long-range morphology of the films. These observations confirm that variations in the Raman intensity were not the result of roughness fluctuations in the film and substrate (see Figure S3 in supplementary information). From Fig. 3 it is clear that the anatase/brookite domain sizes are on the micron scale for the control sample deposited directly on glass, and become substantially larger for films deposited on oxygen-rich sputtered seed layers. Once the anatase domain size has reached a critical value, the likelihood of a continuous anatase area, which extends the length of the sample, becomes very high. Thus, an increase in the domain size is likely responsible for the excellent electrical properties of the sputtered TNO heterostructures (see supplementary – Table S1 and Figure S4).

### *Ex-situ* and Hall effect electrical transport measurements

**Figure 4:**
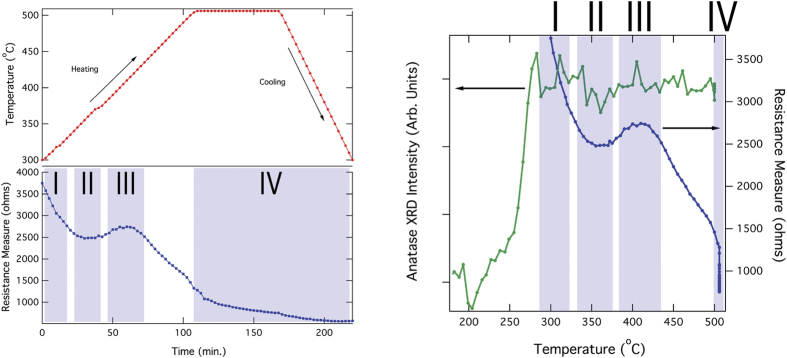
*In –situ* conductivity measurements plotted vs. annealing temperature and time. Temperature dependence of anatase x-ray intensity compared with film resistance.

An *ex-situ* annealing experiment was performed which measured the resistance of a TNO film (deposited on a oxygen rich seed layer) as a function of temperature under a reducing gas environment (5% H_2_ by vol. bal. Ar). The aim of this experiment was to establish a complete conductivity profile as a function of the anneal temperature. The data was collected in traditional 4-point probe fashion with four collinear contacts and the results are plotted in Fig. 4. During the initial stages of annealing, the film resistance begins to decrease around 300 °C. A second, more pronounced decrease in the resistance occurs once the sample attains the anneal hold temperature of 500 °C, where it dwells for 1.5 hrs. This data is directly correlated with the sample resistivity, via a geometric factor associated with the sample and contact geometry, and was used to identify annealing conditions to examine the nature of this resistance trend in more detail via Hall analysis (see Table S1). The Hall analysis addresses a potential pitfall of the *ex-situ* annealing experiments. While in the tube furnace, the sample is free-floating and is not in direct contact with the alumina tube. Despite the slow ramp rate (1 °C/min), the sample may not have reached a true thermal equilibrium with the annealing environment. Therefore, the annealing experiments were used to identify four temperature regions of interest that require further investigation. Hall measurements were then subsequently conducted on 140 nm thick TNO films deposited on top of sputtered-seed layers (5 nm thick). Each of these samples was annealed immediately post-deposition in the vacuum of the sputtering system to a temperature within each region of interest for 2 hrs. Based on the Hall data, the initial decrease in resistivity is likely the result of niobium dopant activation. The “hump” in the sample resistivity at around 325 °C appears to be the result of a change in the mobility. The observed double minimum in the mobility around this temperature is a curious result, as the XRD for all seed layer samples shows an absence in structural changes after the initial crystallization. However, this lack of structure change could be masked by the limitations of our XRD instrument. Bulk TNO films deposited on thinner sputtered seeds outperformed (conductivity) all other seed layer types regardless of thickness. Whilst higher temperatures produced films with better electrical properties given a constant annealing time, it is currently unknown if a sufficiently long anneal at lower temperatures will produce films with equivalent resistivity values. As a sanity check, the transmission/reflection characteristics of our 5% Nb doped TiO_2_ samples were tested and a transparency between 60–80% was obtained even for the most conductive samples (see supplementary – Figure S5). Hall measurements were also conducted on TNO films deposited on ALD seed layers. In this case, the temperature was maintained at a constant 540 °C and the annealing time was varied. Optimum performance of these materials is achieved with an anneal lasting approximately 2 hrs. If the duration of this anneal is increased substantially (18 hrs), the performance of these samples is worsened. This dip in electrical properties is the result of a mobility decrease at the long anneals times.

### Design of experiments

**Figure 5:**
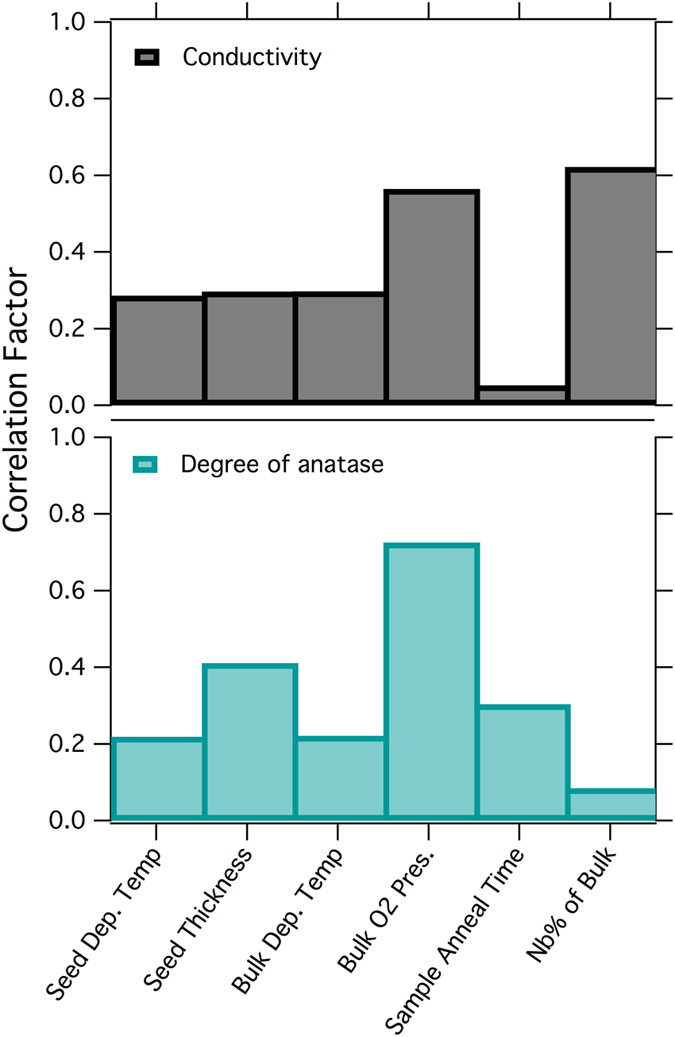
Design of experiments results plotting the film/seed deposition parameters against the figures of merit.

Correlation factors calculated from the analysis of the design of experiments are plotted in Fig. 5. The two most important parameters in achieving films with a high degree of anatase are sputtering on thicker (30 nm) seed layers and deposition of the bulk film in a high oxygen partial pressure (PO_2_). The major factors impacting conductivity are bulk deposition in low PO_2_ and Nb content. 10% Nb doped TiO_2_ films were found to be far less conductive than their 5% Nb counterparts despite being annealed at the same temperature and equivalent durations.

This is likely due to the decrease in doping efficiency, increased dopant-dopant association, and increased dopant-scattering with increasing Nb content (above 6%)^7^. The doping concentration in the bulk film showed no correlation with anatase phase formation. Instead, O_2_ pressure during the deposition of the bulk film was highly correlated and associated with the formation of the anatase phase. However, samples produced with bulk layers deposited in a high PO_2_ tend to have relatively poor electrical properties. We speculate that low oxygen pressure during deposition results in large quantities of oxygen vacancies in the bulk lattice. In order to preserve charge neutrality, these vacancies are compensated with electronic defects. Thus oxygen-rich films essentially quench these additional electronic carriers ultimately resulting in high resistivity. Additionally, oxygen rich conditions increase the formation enthalpy of Nb^+^_Ti_ donors, while the compensating Ti vacancies form with higher probability, and consequently the effective dopant activation is determined by the oxygen content of the as-grown material^22^. Thus, large anatase intensity is not necessarily a good indicator of a highly conductive film. Nonetheless, our results indicate that the mechanism behind the improvement in film properties is the stabilization of the anatase phase in the seed layer, which subsequently acts as a nucleation center for the bulk film crystallization during annealing. The bulk film must be oxygen deficient for high conductivities to be achieved. Additionally, these results confirm previous hypothesize regarding the role of the seed layer on the enhancement of structural and electrical properties of TNO heterostructures^16^.

## Conclusion

The impact of an underlying interfacial seed layer was clarified by carefully examining the dependency of bulk TNO film properties (phase purity, resistivity) on key seed/deposition parameters. This careful analysis relied on Raman spectroscopy, which allowed us to observe the structure and spatial distribution of minor impurity phases that were difficult to resolve with conventional XRD experiments. Our results suggest a key guiding principle to achieve films possessing high phase purity and high electrical conductivity is related to the oxygen content in both the seed and bulk layers. Fabrication of low resistivity TNO bilayer films on glass is realized for heterostructures consisting of an oxygen-rich base layer immediately followed by an oxygen-deficient bulk layer. In this way, detrimental titania polymorphs phases are suppressed while dopant activation occurs at moderate temperatures.

### Experimental

Corning Eagle^2000^ (E2K) glass slides were prepared by ultrasonic cleaning while soaking in acetone and isopropyl alcohol solvents (10 minutes each). The films studied in this work were deposited using the ALD and sputtering methods. To deposit the films, the substrates were alternately exposed to the Ti precursor and oxygen precursor In order to ensure layer-by-layer growth, the reaction zone was purged after each precursor pulse. The substrate temperatures used for deposition ranged from 100 to 300 °C.

Our sputtering targets were all 2-inch diameter disks with composition of Ti_0.90_Nb_0.1_O_2_, Ti_0.95_Nb_0.05_O_2_, and undoped TiO_2_ (99.9% purity). The base pressure achieved prior to each deposition was 5 × 10^−6^ Pa. A mixture of Ar and O_2_ at various flow rate ratios maintaining a total system pressure of 1.0 Pa was utilized during deposition. The RF power applied to the target was kept constant at 75 W. Preceding each deposition, the target surface was first sputter-cleaned in pure Ar for several minutes and subsequently pre-sputtered for 10 min using the same gas flow ratio and total pressure as for the bulk film. The as-deposited films (when amorphous), were crystallized by annealing in the deposition chamber under vacuum (no gas flow). The substrate temperature during deposition/annealing was confirmed using a thermocouple under the same pressures achieved during post treatment processes.

We first deposited a variety of seed layers directly on E2K glass by sputtering and atomic layer deposition (ALD) and subsequently grew thick TNO layers (also by sputtering) with varying oxygen content (typically O_2_ deficient). Samples based entirely on sputtering were performed in the same chamber, thus eliminating the need to break vacuum. For the sputtered seed layers, we elected to vary a number of parameters including seed thickness (5–30 nm), doping concentration (undoped, 5% Nb, and 10% Nb), and oxygen content (5–50% flow rate O_2_). Pure TiO_2_ ALD seeds were grown incorporating several additional factors (precursor chemistry, crystallinity, and annealing duration).

The figures of merit were represented by electrical conductivity and anatase signal intensity from Raman spectroscopy. Note, due to fluctuations in the anatase signal at high magnifications (100X), we opted to reduce the magnification power to 20X in order to sample a larger portion of the film surface. This way, the microscopic variations in the signal intensity are averaged out in a single spot scan. Typically, correlation factors range from +1 to −1, and values near these endpoints indicate strong correlations (positive and negative, respectively) between the growth parameter and the figure of merit. Values approaching zero imply little/no association. In this situation, we have calculated and plotted the absolute value of these correlation factors, and are only interested in growth parameters that result in a large interdependence with the figures of merit.

For the x-ray annealing experiments, all samples were heated at 1 C/min to a maximum temperature of 500 °C in flowing N_2_ while simultaneously acquiring diffraction data. N_2_ was used in order to decrease the oxygen partial pressure during the experiment in order to more accurately represent the annealing conditions in the sputter system vacuum chamber. The samples dwelled at the highest temperature for several hours (~8) to measure changes in the crystal phases present at longer annealing times.

Conductivity measurements were conducted using a standard 4-point probe setup. Crystallographic structure and orientation were characterized by X-ray diffraction (XRD) measurements using a two-dimensional detector (Bruker, D8 Discover with GADDS) and Raman spectroscopy. Film smoothness and long-range characteristics were examined by field emission scanning electron microscopy (FESEM) and atomic force microscopy (AFM). Film thickness was determined using spectroscopic ellipsometry and independently confirmed with FESEM. In order to determine the crystallization temperatures as a function of seed layer, we conducted XRD hot-stage experiments under N_2_ flow^23^,^24^,^25^,^26^,^27^.

## Additional Information

**How to cite this article**: Nikodemski, S. *et al.* The Role of Nanoscale Seed Layers on the Enhanced Performance of Niobium doped TiO_2_ Thin Films on Glass. *Sci. Rep.* **6**, 32830; doi: 10.1038/srep32830 (2016).

## Supplementary Material

Supplementary Information

## Figures and Tables

**Table 1:** Seed layer and Bulk TNO layer deposition experimental details.

Deposition variables	ALD seed layers	Sputtered seed layers	Bulk TNO layers on seed layers
Ti precursor	TiCl_4_, Ti[OCH(CH_3_)_2_]_4_	—	—
Oxidizer	H_2_O, H_2_O_2_	—	—
Dep. temp (^o^C)	100, 300	RT	RT, 550
Anneal temp (^o^C)	—	550	200–550
Anneal time (hrs.)	—	2	2–18
Oxygen flow rate ratio (%)	—	0–50	0–30
Thickness (nm)	5, 30	5, 30	140
Niobium content (at. %)	0	5, 10	5, 10
