# Supplementary material for: The Role of Nanoscale Seed Layers on the Enhanced Performance of Niobium doped TiO2 Thin Films on Glass
Source: Sci Rep. 2016 Sep 9;6:32830. doi: 10.1038/srep32830 (PMC5017507; doi:10.1038/srep32830)
Supplement: Supplementary Information [file srep32830-s1.doc]

**Supplementary Information**

**The Role of Nanoscale Seed Layers on the Enhanced Performance of Niobium doped TiO_2_ Thin Films on Glass**

*Stefan Nikodemski, Arrelaine A. Dameron, John D. Perkins, Ryan P. O’Hayre, David S. Ginley, and Joseph J. Berry^*^*

Stefan Nikodemski and Ryan P. O’Hayre

Department of Metallurgical and Materials Engineering, Colorado School of Mines, 1500 Illinois Street, Golden, CO 80401, USA

Arrelaine A. Dameron, John D. Perkins, David S. Ginley, and Joseph J. Berry

National Renewable Energy Laboratory, 15013 Denver W Pkwy, Golden, CO 80401, USA


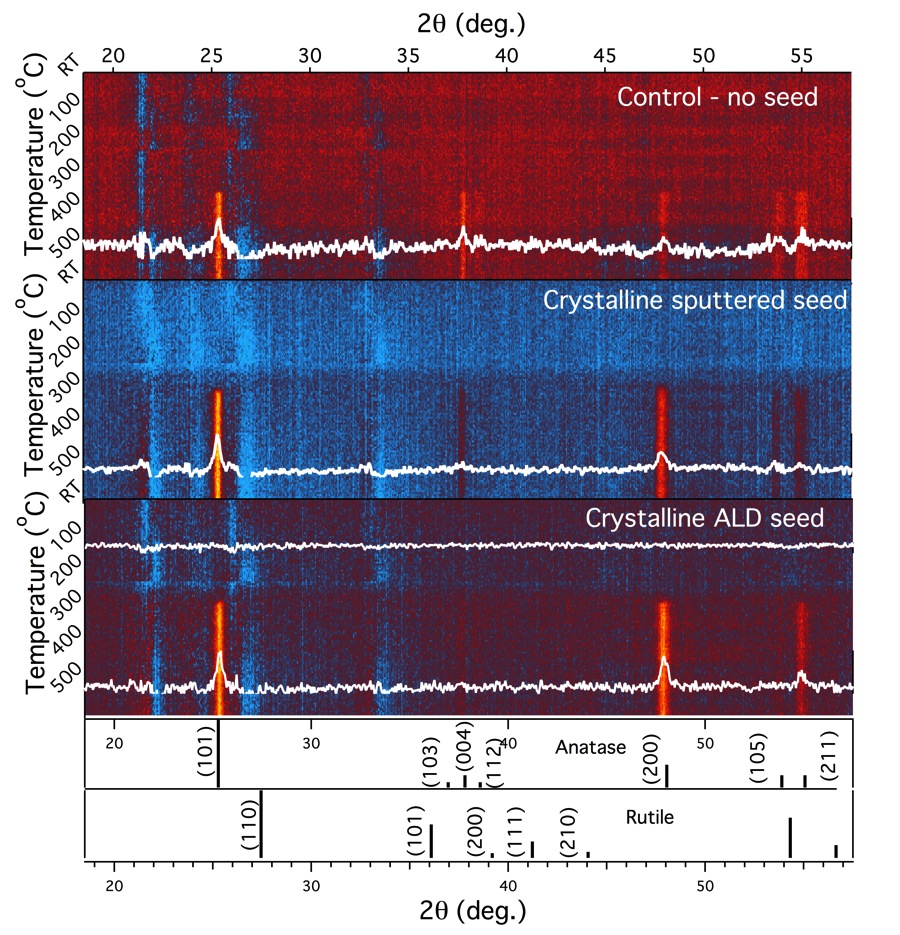


**Figure S1**: Composite image of x-ray diffraction spectra for Nb:TiO_2_ films (deposited on seed layers as well as directly on glass) measured as a function of annealing temperature.


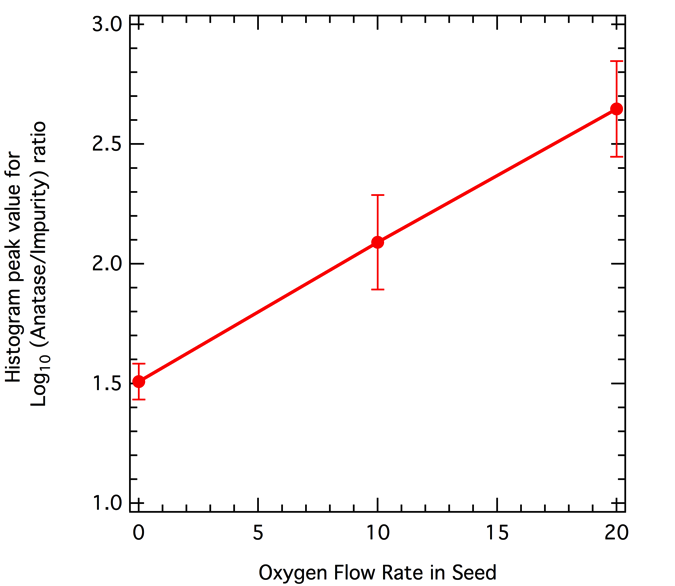


**Figure S2**: Peak value and FWHM of the Raman mapping data (Gaussian fit) histograms for sputtered TNO5 samples with different oxygen flow rates for the seed layer deposition.


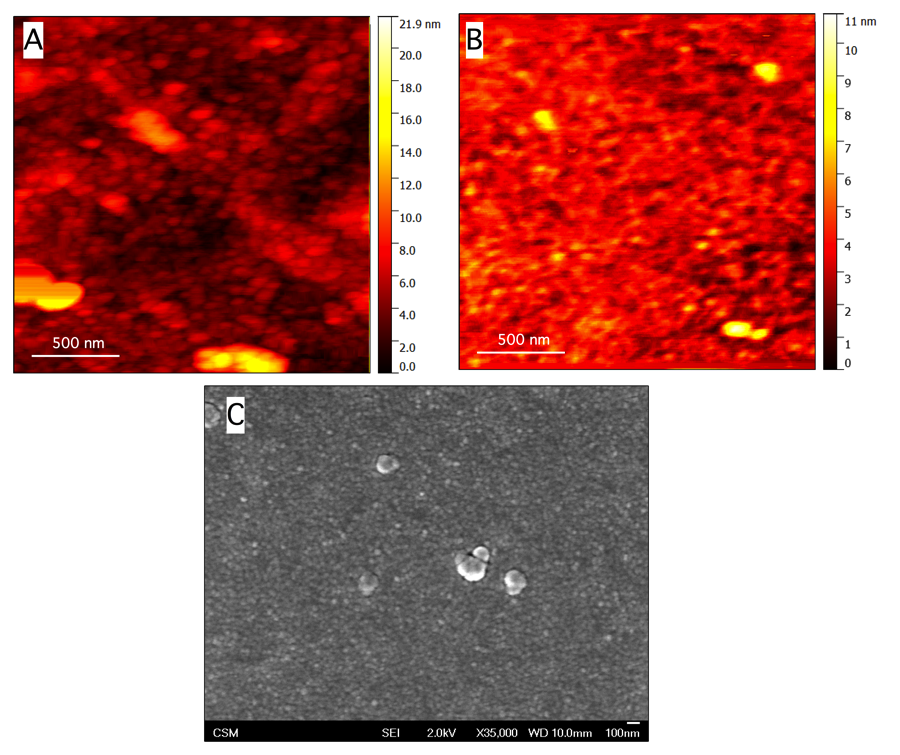


**Figure S3**: AFM and FESEM images showing the surface morphology/roughness after bulk Nb:TiO_2_ deposition. A) ALD seed+bulk TNO layer after dep. B) sputtered seed+bulk TNO film after annealing C) Top view FESEM image of ALD+bulk TNO sample.

**Table S1**: Detailed Hall effect measurements corresponding to regions of interest in *in-situ* annealing

| Annealing  Region | Mobility  (cm^2^ V^-1^ s^-1^ ) | | Carrier Concentration  (cm^-3^) | | | Resistivity  (ohm cm) |
| --- | --- | --- | --- | --- | --- | --- |
| I | | 1 | | 1×10^17^ | 54 | |
| II | | 3.8 | | 1.07×10^21^ | 1.5×10^-3^ | |
| III | | 2.6 | | 1.28×10^21^ | 1.88×10^-3^ | |
| IV | | 4.5 | | 1.39×10^21^ | 1×10^-3^ | |


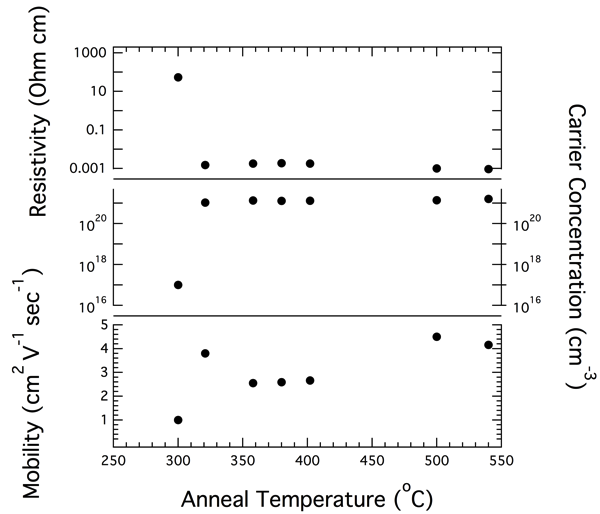


**Figure S4**: Hall effect measurements for a series of individual TNO5 samples deposited on amorphous sputtered seed layers (high oxygen content) annealed to various temperatures inside the vacuum chamber directly after deposition for 2 hrs.


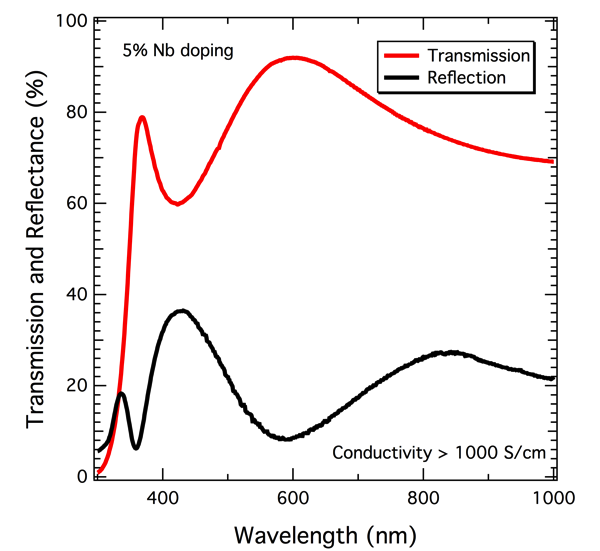


**Figure S5**: Transmission data for high conductivity (>1000 S/cm) sputtered seed layer sample - 5% Nb doped TiO_2_ composition on E2K glass.
